# Assessment of the relationship of serum liver enzymes activity with general and abdominal obesity in an urban Bangladeshi population

**DOI:** 10.1038/s41598-021-86216-z

**Published:** 2021-03-23

**Authors:** Nurshad Ali, Abu Hasan Sumon, Khandaker Atkia Fariha, Md Asaduzzaman, Rahanuma Raihanu Kathak, Noyan Hossain Molla, Ananya Dutta Mou, Zitu Barman, Mahmudul Hasan, Rakib Miah, Farjana Islam

**Affiliations:** Department of Biochemistry and Molecular Biology, Shahjalal University of Science and Technology, Sylhet, 3114 Bangladesh

**Keywords:** Endocrine system and metabolic diseases, Obesity

## Abstract

Obesity is a global health concern because of its increasing trend both in developed and developing countries. A limited number of studies have evaluated the association of liver enzymes with both general and abdominal obesity in the general population; data for the Bangladeshi population are not available yet. This study aimed to assess the relationship of serum liver enzymes activity with both general and abdominal obesity in Bangladeshi adults. In total, 540 blood samples were obtained from the participants (388 males and 152 females) and analyzed for serum levels of ALT, AST, GGT, and ALP using standard methods. General obesity was defined as body mass index (BMI) ≥ 27.5 kg/m^2^ and abdominal obesity was defined as waist circumference (WC) ≥ 90 cm in males and ≥ 80 cm in females. The relationship between liver enzymes and obesity was evaluated by multivariate logistic regression models. Overall, 58% of participants in the general obesity group and 55% of the participants in the abdominal obesity group had at least one or more elevated levels of liver enzymes. The prevalence of elevated liver enzymes was significantly higher in the obesity group compared to the normal BMI and WC groups (*p* < 0.05 for all cases). The mean level of serum ALT, AST and GGT were significantly higher in the obesity group than the normal BMI group (*p* < 0.05). In the WC groups, mean AST and GGT were significantly higher in the obesity group compared to the normal group (*p* < 0.05). In regression analysis, serum levels of ALT showed an independent and significant association with general obesity, whereas, serum GGT showed a significant association with both general and abdominal obesity. In conclusion, a high prevalence of elevated liver enzymes was observed among participants included in the present study. Of the four enzymes, serum GGT was independently associated with both general and abdominal obesity. Further studies are required to understand the complex relationship between liver enzymes and obesity in the general population.

## Introduction

Both general and abdominal obesity are global health concerns because of their increasing trend both in developed and developing countries^1,2^. According to the World Health Organization (WHO), about 39% of adults were overweight and 13% of the world's adult population were obese in 2016^3^. In epidemiological studies, waist circumference (WC) and waist-hip ratio (WHR) are used as measures of abdominal/central obesity and body mass index (BMI) is used as a measure of general obesity. In 2010, the WHO estimated overweight (BMI ≥ 25 kg/m^2^) prevalence was 8.4% in Bangladeshi male subjects (aged > 15)^4^. In another study, the prevalence of general and abdominal obesity was reported as 26.2% and 39.8%, respectively in rural adults in Bangladesh^5^. Both general and abdominal obesity has been found to be associated with a number of metabolic disorders including type 2 diabetes, hypertension and cardiovascular disorders^6,7^.

The prevalence of general and abdominal obesity has been increased in the last few decades due to unhealthy dietary habits, less physical activity and increased sedentary lifestyles^8^. Obese, diabetic, hypertensive and married individuals are at higher risk of having non-alcoholic fatty liver disease (NAFLD) than others^9^. Increased levels of WC and BMI have been found to be independently related to NAFLD and people with abdominal obesity have an increased risk of NAFLD than subjects with general obesity^8^. Some epidemiological studies and meta-analyses demonstrated that abdominal obesity is an important predictor of metabolic disorder along with general obesity^10–12^. In earlier studies, abdominal obesity has also been considered as an indispensable component of metabolic syndrome rather than general obesity^13^. This suggests that abdominal obesity should not be ignored as a contributing risk factor for NAFLD^14^. Even, abdominal obesity is a considerable predictor for NAFLD in subjects with normal body weight^15,16^.

The four serum enzymes that are generally used in assessing liver functions are alanine and aspartate aminotransferase (ALT, AST), alkaline phosphatase (ALP) and γ-glutamyltransferase (GGT)^17^. Serum ALT is considered a particular marker for hepatic dysfunction and is found mainly in this organ^18,19^, while serum GGT is present in most cell surfaces and shows a high activity in the kidney and liver^20^. Previously, some studies have been carried out to evaluate the relationship of ALT and GGT with obesity^21–29^. A positive correlation was found for abdominal obesity with ALT and GGT in previous studies^26,30^. Most of the earlier studies examined the associations considering a less number of liver enzymes and reported inconsistent results. Up to now, a few number of studies evaluated the relationship of the maximum number of hepatic enzymes level with both abdominal and general obesity in the general adult population. So far, no study has been carried out to assess the relationship between liver enzymes and obesity in the Bangladeshi population. In the present study, we aimed to evaluate the relationships of serum liver enzymes activity with both general and abdominal obesity in Bangladeshi general adults.

## Methods

### Study design and participant selection

This study was a cross-sectional design, conducted between October 2017 and September 2018 at the Department of Biochemistry and Molecular Biology of Shahjalal University of Science and Technology, Bangladesh. In total, 540 participants (388 males and 152 females) were enrolled in the study. The participants consisted of young adult students, the academic and non-academic staffs from different departments of the university and general adults from local city areas. The participants were randomly selected. The inclusion criteria were both sexes, aged > 18 years, not having serious sickness and willing to take part in the study. Subjects with a history of hepatotoxic drug intake, and alcohol intake currently or in the past and self-reported proof of acute or chronic hepatitis, were excluded from the study. Lactating mothers, pregnant women, and the subjects with missing demographic data were also excluded from the study. All participants provided written informed consent and the study was approved by the Internal Ethical Review Board, Department of Biochemistry and Molecular Biology, School of Life Sciences, Shahjalal University of Science and Technology, Bangladesh (Reference ID. 02/BMB/2019). All steps of the methods section were conducted following the appropriate guidelines and regulations.

### Data collection

Data collection procedure included fulfilling a brief questionnaire on anthropometric and demographic measurements, smoking status and physical activity. The anthropometric measurements were performed with the help of trained medical personnel following standard procedure described elsewhere^31–36^. In brief, anthropometric data, such as weight, height, waist and hip circumference were measured with the participants wearing no shoes and light clothes^37^. Body weight was taken to the nearest 0.1 kg by modern electronic digital LCD weighing scales (Beurer 700, Germany)^37^. Height was taken to the nearest 0.1 cm while the participants stood in the erect posture. Body mass index (BMI) was reported as weight in kilograms (kg) divided by height in meters squared (m^2^). Waist circumference (WC) was measured using general tape that was placed midway between the lower margin of the last palpable rib and iliac crest on the mid-axillary line ^37,38^. Hip circumference (HC) was measured at the largest circumference of the buttocks^37^. Waist-to-hip ratio (WHR) was then obtained by dividing the WC by the HC.

### Sample collection and biochemical analysis

After an overnight fast, about 5 ml of the blood sample was collected from each participant. Blood samples were centrifuged and stored at − 80 °C until biochemical analysis. Serum levels of ALT, AST, GGT and ALP were determined by kinetic methods. Fasting blood glucose, total cholesterol (TC), and triglycerides (TG), were measured by colorimetric methods^39^. All biochemical parameters were measured using commercial diagnostic kits (Human Diagnostic, Germany, except GGT from Vitro Scient, Egypt)^39^. All the biochemical parameters were analyzed in a biochemistry analyzer (Humalyzer 3000, USA).

### Definitions

BMI was divided into three groups: normal weight (18.5–23.0 kg/m^2^), overweight (23.1–27.5 kg/m^2^), and obesity (≥ 27.5 kg/m^2^)^40^. Abdominal obesity was defined as a WC ≥ 80 cm for females and ≥ 90 cm for males^41^. Abnormal or elevated hepatic enzymes were defined as least one or more measurement of: ALT > 45 U/L in males/ > 34 U/L in females, AST > 35 U/L in males/ > 31 U/L in females, GGT > 55 U/L in males/ > 38 U/L in females^42^ and ALP > 129 U/L in males/ > 104 U/L in females^43^. Smoking status was classified as current smokers or non/ex-smokers. Physical activity was categorized as light (easeful office jobs or little housework), moderate (general walking, swimming, and household goods cleaning) and heavy/adequate (lifting, carrying, jogging and/or sports).

### Statistical analysis

Results are presented as means ± SD. Pearson’s correlation coefficient was used to measure the correlation between liver enzymes and baseline variables. The normality of the data was assessed before choosing the parametric tests. Independent sample t-test (Student’s t-test) and one-way ANOVA with Dunnett’s Post Hoc Test were applied to compare the variables between sex groups and BMI groups, respectively. The Chi-square test was used to compare the prevalence of elevated enzymes within or between the BMI and WC groups. The relationship of liver enzymes with general and abdominal obesity was evaluated by multivariate logistic regression models. Both general and abdominal obesity was categorized as yes (presence) and no (absence), other variables were considered as a continuous variable. In regression analysis, obesity (yes) was taken as the dependent variable and liver enzymes as the independent variable. Three models were applied in the regression analysis. In model 1, age and sex were adjusted. In model 2, TG and TC were further adjusted and in model 3, smoking status and physical activity were additionally adjusted. IBM SPSS software, version 23 was used for data analysis. A p-value < 0.05 was considered statistically significant.

## Results

### Baseline characteristics in the sex groups

The demographic and baseline information of the participants are summarized in Table 1. Out of 540 participants, 388 were males and 152 were female subjects. Overall, the mean age was 40.1 ± 15.7 years with no significant difference between male (39.3 ± 16.0 years) and female (42.3 ± 14.6 years) subjects. There was no significant difference for BMI, WC, HC and WHR in the male–female groups. Of the liver enzymes, the mean level of AST was significantly higher in females than in the male participants (p < 0.01). Female subjects had a higher mean concentration of serum glucose (p < 0.001) than the male participants. The average concentration of serum TG was significantly higher in males, whereas, TC was higher in females (p < 0.05).

**Table 1:** Baseline characteristics of participants.

Variables	Total	Male	Female	P-value
*N*	540	388	152	-
Age (years)	40.1 ± 15.7	39.3 ± 16.0	42.3 ± 14.6	0.217
BMI (kg/ m^2^)	24.7 ± 3.9	24.6 ± 3.6	24.7 ± 4.6	0.895
WC (cm)	86.6 ± 10.5	86.7 ± 10.1	86.5 ± 11.6	0.882
HC (cm)	92.8 ± 8.2	93.1 ± 7.8	92.1 ± 9.4	0.441
WHR	0.93 ± 0.07	0.93 ± 0.06	0.94 ± 0.08	0.463
Glucose (mg/dL)	104.7 ± 27.7	97.3 ± 15.6	130.2 ± 42.1	0.000
ALT (U/L)	30.4 ± 15.0	30.3 ± 13.5	30.6 ± 19.2	0.916
AST (U/L)	30.9 ± 13.5	29.4 ± 11.1	35.1 ± 18.5	0.008
GGT (U/L)	31.7 ± 24.5	33.2 ± 26.8	26.8 ± 14.6	0.107
ALP (U/L)	100.4 ± 44.8	101.4 ± 45.4	97.4 ± 43.5	0.583
TG (mg/dL)	190.9 ± 123.7	205.1 ± 127.6	150.9 ± 103.2	0.010
TC (mg/dL)	212.5 ± 96.1	201.7 ± 88.5	241.7 ± 110.0	0.012

Data are presented as mean ± SD. P-values are obtained from the independent sample t-test in comparison between male–female groups.

### Baseline characteristics of the study subjects in the BMI and WC groups

**Figure 1:**
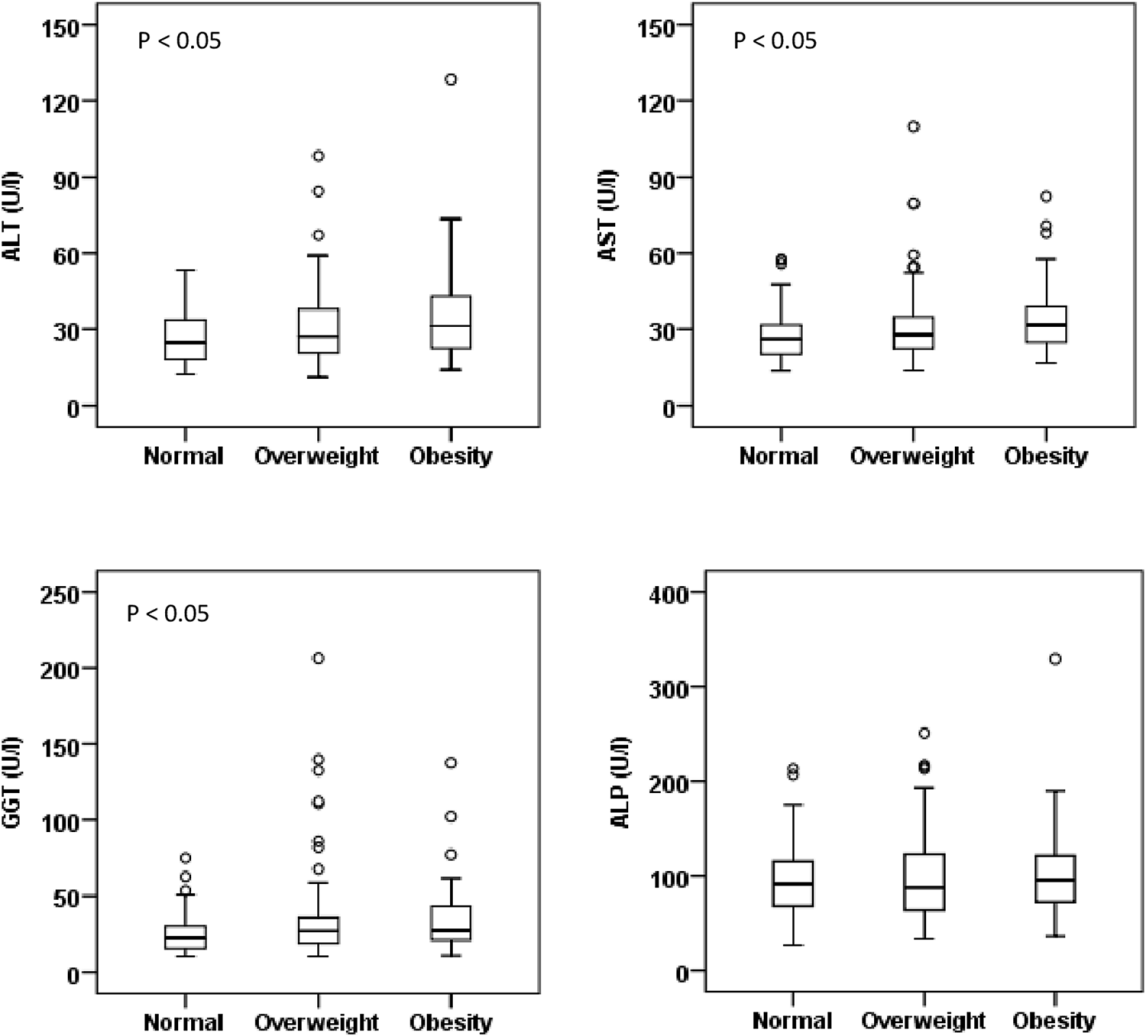
Levels of serum liver enzymes in the BMI groups (n = 173 in normal, n = 259 in overweight and n = 108 in obesity group). P-values are obtained from one-way ANOVA (Dunnett’s Post Hoc Test). *P < 0.05 when the mean level of liver enzymes in the normal group is compared to the obesity group.

**Figure 2:**
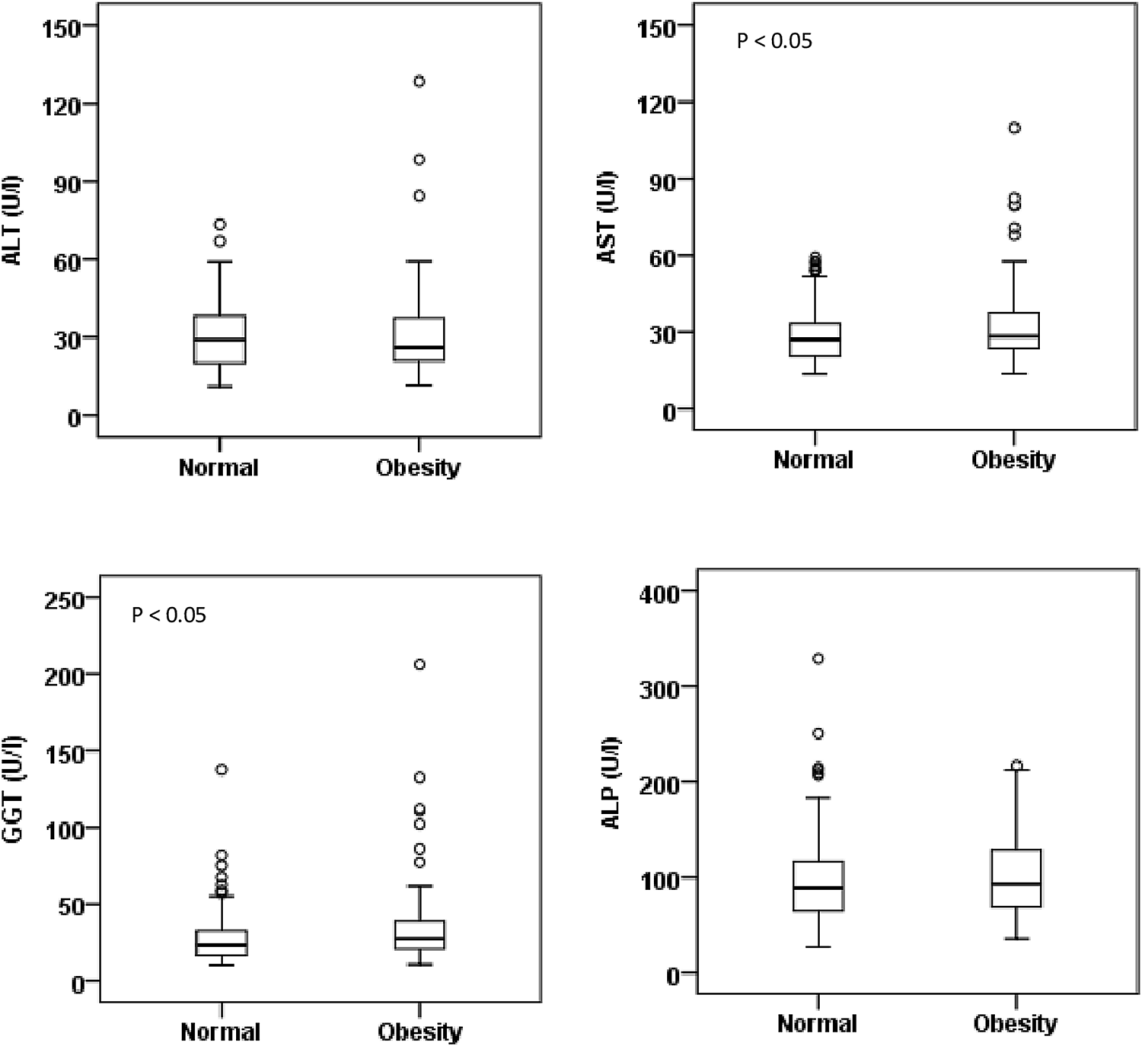
Levels of serum liver enzymes in the WC groups (n = 296 in normal and n = 244 in obesity group). P-values are obtained from independent sample t-test. *P < 0.05 when the mean level of liver enzymes in the normal group is compared to the obesity group.

**Figure 3:**
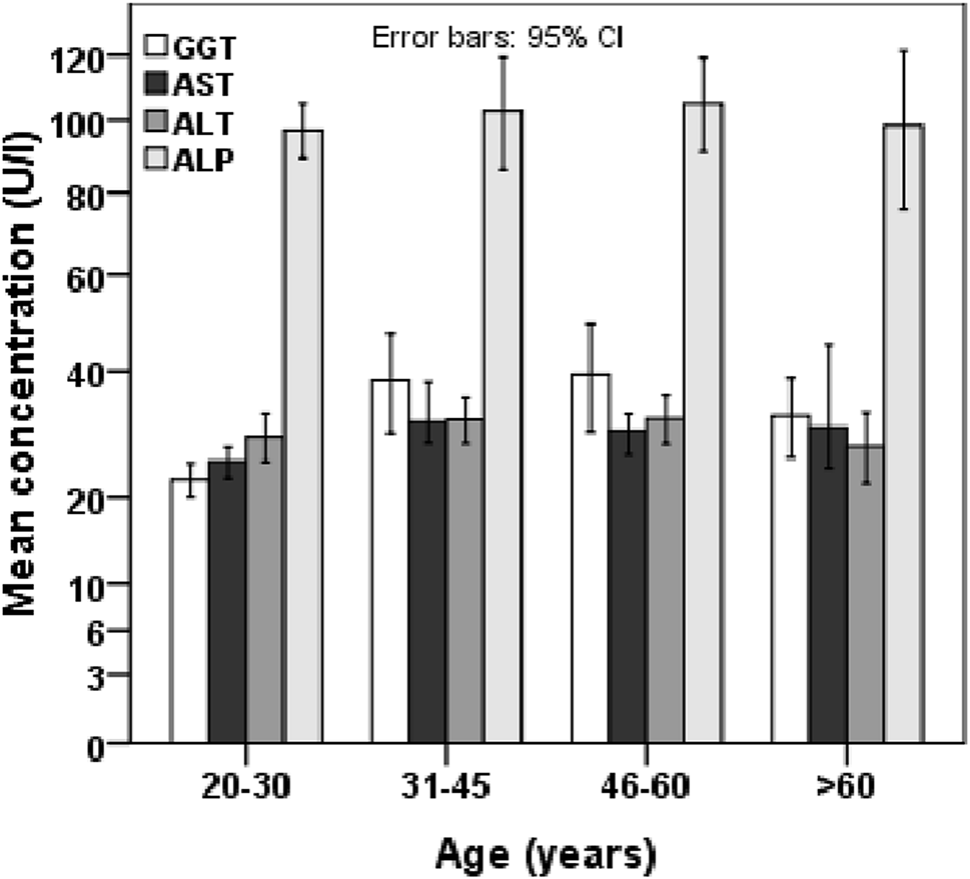
Levels of serum liver enzymes in the different age groups (n = 210 in 20–30 years, n = 134 in 31–45 years, n = 134 in 46–60 years and n = 62 in > 60 years group). The levels of liver enzymes in the age groups are compared by one way ANOVA.

Baseline characteristics and levels of liver enzymes of the study subjects in different BMI and WC groups are presented in Table 2 and Fig. 1. The prevalence of general and abdominal obesity was 20% (n = 108) and 45.2% (n = 244) in the BMI and WC groups, respectively. There was a significant difference in the mean level of BMI, WC, HC and WHR between and within the BMI groups. The mean level of serum ALT, AST and GGT was significantly higher in the obesity group compared to the normal BMI group (p < 0.05). The serum level of ALP did not show significant differences between or within the BMI groups. In the WC groups, the mean level of serum AST and GGT were significantly higher in the obesity group compared to the normal group (p < 0.05) (Table 2 and Fig. 2). The average level of liver enzymes was found to be higher in the 31–60 years age group compared to other age groups (Fig. 3). A decreasing trend for the levels of liver enzymes was observed among the participants aged above 60 years. A significant difference was also observed for serum glucose concentration between the normal and obese individuals in the BMI (p < 0.01) and WC (p < 0.05) groups. However, no significant difference was found for serum TG and TC within or between the BMI groups, whereas, in the WC groups, the mean level of serum TC was significantly higher in the obese group (p < 0.01).

**Table 2:** Baseline characteristics of study participants in the BMI and WC groups.

Variables	Body mass index (BMI)				Waist circumference (WC)		
	Normal	Overweight	Obesity	P-value	Normal	Obesity	P-value
*N*	173	259	108	-	296	244	-
Sex (m/f)	121/52	200/59	67/41	-	245/51	143/101	-
Age (years)	37.8 ± 17.4	40.7 ± 15.3	42.2 ± 13.7	0.299	37.3 ± 16.2	43.5 ± 14.4	0.003
BMI (kg/ m^2^)	20.5 ± 1.8	25.1 ± 1.2	30.5 ± 2.7	< 0.001	22.7 ± 2.8	27.1 ± 3.7	< 0.001
WC (cm)	78.1 ± 8.3	88.1 ± 7.6	97.7 ± 8.5	< 0.001	79.6 ± 6.8	95.3 ± 7.3	< 0.001
HC (cm)	86.8 ± 6.2	93.5 ± 6.0	101.5 ± 7.9	< 0.001	88.2 ± 6.1	98.5 ± 6.9	< 0.001
WHR	0.90 ± 0.06	0.94 ± 0.06	0.96 ± 0.06	< 0.001	0.90 ± 0.06	0.97 ± 0.06	< 0.001
Glucose (mg/dL)	98.9 ± 13.5	103.1 ± 21.2	125.8 ± 54.1	0.006	99.4 ± 13.3	114.8 ± 42.1	0.010
ALT (U/L)	26.7 ± 10.5	30.7 ± 15.0	35.6 ± 19.8	0.011	29.6 ± 12.5	31.4 ± 17.8	0.411
AST (U/L)	27.6 ± 9.8	31.3 ± 14.7	34.7 ± 14.7	0.024	29.0 ± 10.7	33.2 ± 16.1	0.027
GGT (U/L)	24.9 ± 12.7	34.9 ± 29.8	35.4 ± 24.7	0.022	27.7 ± 17.5	35.5 ± 28.8	0.019
ALP (U/L)	96.2 ± 38.9	99.6 ± 45.5	104.2 ± 50.5	0.660	99.3 ± 47.9	101.7 ± 40.9	0.705
TG (mg/dL)	160.9 ± 85.6	197.6 ± 135.8	213.9 ± 127.1	0.071	179.5 ± 109.5	204.6 ± 138.4	0.178
TC (mg/dL)	201.6 ± 87.8	223.6 ± 93.4	234.6 ± 109.8	0.207	190.1 ± 83.7	237.4 ± 104.0	0.001

Data are presented as mean ± standard deviation (SD). P-values are obtained from the independent sample t-test in the WC groups and one-way ANOVA in the BMI groups.

### Prevalence of elevated liver enzymes

Approximately, 58% of participants in the general obesity group and 55% of the participants in the abdominal obesity group had at least one or more elevated hepatic enzymes. The prevalence of all elevated liver enzymes (except ALP) in the BMI and WC groups are shown in Table 3. The differences in the prevalence of elevated liver enzymes within and between the BMI groups were significant (p < 0.01). For ALP, this elevation was significant when the prevalence in the obesity group was compared with the normal and overweight group (p < 0.05). In the WC based groups, the prevalence of elevated hepatic enzymes was significantly higher in the obesity group compared to the normal group (p < 0.05 for all cases).

**Table 3:** Prevalence of elevated liver enzymes in the BMI and WC groups.

	Body mass index (BMI)				Waist circumference (WC)		
	Normal (%)	Overweight (%)	Obesity (%)	P-value for trend	Normal (%)	Obesity (%)	P-value
ALT	11.5	16.5	26.5	< 0.01	13.2	19.8	< 0.05
AST	20.0	28.1	48.5	< 0.01	25.7	31.1	< 0.05
GGT	7.5	13.5	34.6	< 0.01	9.4	19.2	< 0.01
ALP	29.6	26.7	40.7	< 0.05	21.9	33.3	< 0.05

Chi-square test was applied to derive the p-values.

### Association of ALT and GGT with general and abdominal obesity

Taking obesity as the dependent variable and liver enzymes as the independent variable, multivariate logistic regression was done to evaluate the relationship between liver enzymes and obesity. Serum levels of ALT showed a significant association with only general obesity in the regression models, whereas, GGT showed a significant relationship with both general and abdominal obesity (Table 4). In all models of the regression analysis, serum GGT showed a stronger association with obesity than the other three liver enzymes.

**Table 4:** Association of liver enzymes with general and abdominal obesity.

		ALT		AST		GGT		ALP	
		OR (95% Cl)	P-value	OR (95% Cl)	P-value	OR (95% Cl)	P-value	OR (95% Cl)	P-value
*General obesity*									
Model 1		1.04 (1.01–1.07)	0.006	1.04 (1.00–1.07)	0.024	1.03 (1.01–1.06)	0.016	1.00 (0.99–1.01)	0.376
Model 2		1.04 (1.01–1.07)	0.033	1.04 (0.99–1.08)	0.053	1.05 (1.01–1.09)	0.015	1.00 (0.99–1.01)	0.999
Model 3		1.04 (1.01–1.07)	0.046	1.04 (0.99–1.08)	0.072	1.05 (1.01–1.09)	0.020	1.00 (0.99–1.01)	0.936
*Abdominal obesity*									
Model 1		1.01 (0.99–1.03)	0.412	1.01 (0.99–1.04)	0.301	1.02 (1.00–1.03)	0.021	1.01 (0.99–1.01)	0.580
Model 2		1.02 (0.99–1.05)	0.157	1.02 (0.99–1.05)	0.259	1.03 (1.01–1.06)	0.011	1.00 (0.99–1.01)	0.783
Model 3		1.02 (0.99–1.05)	0.213	1.02 (0.99–1.05)	0.236	1.04 (1.01–1.07)	0.014	1.00 (0.99–1.01)	0.747

Multivariate logistic regression analysis was done to assess the relationship of liver enzymes with general and abdominal obesity Dependent variable is obesity (yes) and independent variable is liver enzymes (U/L). Reference category is normal (non-obesity). Model 1: adjusted for age (years) and sex (male and female). Model 2: model 1 + TG and TC (mg/dL). Model 3: model 2 + smoking status, and physical activity. OR, odds ratio; CI, confidence interval.

## Discussion

In the present study, serum ALT was significantly associated with general obesity, whereas GGT was associated with both general and abdominal obesity. To our knowledge, this is the first report that has added information regarding the association of liver enzymes with obesity in a Bangladeshi adult cohort.

The prevalence of general and abdominal obesity was about 20% and 45% in the BMI and WC groups, respectively. The study subjects were mainly recruited from the urban regions, which might be a reason behind this relatively high prevalence of obesity. A high prevalence of obesity in Bangladeshi urban population has been described in a recent review^44^. Approximately, 58% of participants in the general obesity group and 55% of the participants in the abdominal obesity group had at least one or more elevated levels of liver enzymes. The prevalence of elevated liver enzymes was significantly higher in the BMI and WC based obesity group. A variation in the elevated levels of hepatic enzymes has been observed between male and female subjects. Elevated hepatic enzymes level was more common in middle than older age among the participants. In middle age group, an elevated ALT level has been found consistently in other studies^23,27,45–47^.

The present study findings are in line with previous studies, where aminotransferase, especially ALT has been shown to be associated with general obesity^21,23,24,26–29^. An association between GGT and obesity has been demonstrated in various studies^21,22,25,48–51^. Our findings and the literature suggest an important role of GGT in the manifestation of hepatic pathology associated with obesity.

A significant correlation has been reported between increased ALT and WC in Korean adults^26^. Among Australian adults, BMI and WC were found to be strongly associated with GGT and ALT^30^. In that study, the increased risk of ALT was about seven times higher among individuals in the obese group, whereas, it was only two times higher among individuals with heavy or moderate alcohol consumption^30^. Moreover, no interactive effects were found between BMI or WC and alcohol consumption on serum ALT and GGT concentrations^30^. Alcohol consumption may also cause hepatic injury and abnormalities in hepatic enzymes concentrations^52^. It is important to mention that consumption of alcoholic drinks is not very usual among the Bangladeshi population except in rare cases due to religious restrictions. Thus, there is less chance to have an effect of alcohol consumption on liver enzyme concentration in Bangladeshi adults. In the Australian population, liver injury is commonly associated with obesity than excessive alcohol use^30^. A study conducted by Stranges et al. supports the role of central adiposity independent from BMI in predicting elevated levels of liver enzymes especially ALT and GGT^53^. In contrast, no effect was observed for decreased BMI on serum ALT and AST levels^54^. In another study, ALT, AST and GGT levels were found to be higher in obese individuals but these liver enzymes did not show any significant correlation with obesity^55^. These inconsistent findings may reflect the differences of population groups between the studies.

The majority of the liver enzyme abnormalities are likely to be because of NAFLD with merely a smaller extent clarified by over alcohol consumption, virus-induced hepatitis or excessive iron load^30^. Malnutrition (undernutrition or overnutrition) is more prevalent in lower to middle-income countries. It has been reported that both malnutrition and visceral/general obesity is associated with NAFLD^56^. Due to increasing rates of obesity, NAFLD was found as the most common cause of liver dysfunction in UK adults^57^.

High values of WC, WHR and BMI have been reported to be associated with NAFLD and subjects with abdominal obesity have a greater risk of fatty liver disease compared to subjects having general obesity^8^. A study demonstrated that abdominal obesity is correlated with much visceral adipose tissue that enhances the portal free fatty acid load flowing to the hepatic cells, and influencing hepatic fat deposition^58^. Visceral adipose tissue is also connected with the degree of liver steatosis in diabetic individuals^59^. The measure of abdominal obesity was found to be correlated with ALT concentrations and cirrhosis-induced mortality or hospitalization at an increased rate than normal BMI^53,60^. Moreover, individuals with both general and abdominal obesity showed an increased risk of developing fatty liver compared to obese persons with no abdominal obesity^15^. Thus abdominal obesity poses a significant health threat than general obesity^8^.

The exact causes of the pathogenesis of NAFLD are not well known yet, although it is proposed that visceral adiposity and insulin resistance may play a significant role in it^61,62^. Moreover, recent evidence suggests that adipokines (such as leptin, adiponectin, and resistin) secreted in adipose mass are closely associated with insulin resistance, obesity, and an early inflammatory stage of NAFLD pathogenesis^63^. Elevated C-reactive protein concentration, a marker of inflammation^64^, is found in NAFLD as an inflammatory agent linked to insulin resistance and also found to associate with metabolic syndrome and elevated ALT concentrations^65–67^. It has also been hypothesized that elevated hepatic enzymes in overweight and obese individuals may result from a combination of hyperlipidemia, hyperinsulinism and reduced antioxidant levels^68^. However, the underlying mechanisms for the elevation of liver enzymes in general and abdominal obesity remain unclear and require further investigations.

The strength of the current study is adjusting known obesity risk factors including age, BMI, physical activities, blood lipid levels and other possible covariates to assess the relationship. However, there were some limitations. Firstly, we measured the liver enzymes only once which do not represent the long-term profile and our study design was cross-sectional, therefore, a temporary sequence could not be inferred. Secondly, we could not rule out the presence of rare liver diseases such as primary biliary cirrhosis and autoimmune hepatitis. Although it is not clear yet that these rare diseases represent the elevation of ALT in the significant number of patients. Thirdly, we used participant’s self-report for some covariate measures in the analysis such as physical activity. Fourthly, the participant’s number was relatively small and enrolled mainly from the urban areas; therefore, the present study findings may not represent the whole Bangladeshi population. However, our study findings could be used as a worthy reference on this topic for future investigation in general adults.

## Conclusions

A high prevalence of elevated hepatic enzymes was observed among study participants. Serum levels of ALT showed a significant association with only general obesity, whereas, GGT showed a significant relationship with both general and abdominal obesity. Of the four enzymes, serum GGT showed the strongest correlations with obesity than the other three liver enzymes, and it may be a better indicator of hepatic pathology associated with general and abdominal obesity. Further studies with larger cohorts from different environments are needed to understand the complex relationship between liver enzymes and obesity in the Bangladeshi population.
